# Highly Efficient* In Vitro* Reparative Behaviour of Dental Pulp Stem Cells Cultured with Standardised Platelet Lysate Supplementation

**DOI:** 10.1155/2016/7230987

**Published:** 2016-09-28

**Authors:** Pasquale Marrazzo, Francesco Paduano, Francesca Palmieri, Massimo Marrelli, Marco Tatullo

**Affiliations:** ^1^Tecnologica Research Institute, Biomedical Section, Crotone, Italy; ^2^Unit of Experimental Surgery, Calabrodental, Crotone, Italy

## Abstract

Dental pulp is an accessible source of multipotent mesenchymal stromal cells (MSCs). The perspective role of dental pulp stem cells (DPSCs) in regenerative medicine demands an* in vitro* expansion and* in vivo* delivery which must deal with the safety issues about animal serum, usually required in cell culture practice. Human platelet lysate (PL) contains autologous growth factors and has been considered as valuable alternative to fetal bovine serum (FBS) in cell cultures. The optimum concentration to be added of such supplement is highly dependent on its preparation whose variability limits comparability of results. By* in vitro* experiments, we aimed to evaluate a standardised formulation of pooled PL. A low selected concentration of PL (1%) was able to support the growth and maintain the viability of the DPSCs. The use of PL in cell cultures did not impair cell surface signature typically expressed by MSCs and even upregulated the transcription of Sox2. Interestingly, DPSCs cultured in presence of PL exhibited a higher healing rate after injury and are less susceptible to toxicity mediated by exogenous H_2_O_2_ than those cultured with FBS. Moreover, PL addition was shown as a suitable option for protocols promoting osteogenic and chondrogenic differentiation of DPSCs. Taken together, our results indicated that PL is a valid substitute of FBS to culture and differentiate DPSCs for clinical-grade use.

## 1. Introduction

Dental pulp stem cells (DPSCs) are multipotent stromal/mesenchymal stem cells (MSCs) representing a source, free of ethical issues, of replacement cells worthwhile in human therapies. From a clinical point of view, it is necessary to avoid any immunological reaction to the xenogenic material; then the investigation on xeno-free* ex vivo* expansion of MSCs is highly encouraged. Many platelet derivates were tested so far in different forms for cell culture to replace animal serum supplementation. Currently, the use of human platelet lysate (PL) in clinical settings led to many therapeutic successes including orthopaedic, periodontal, oral, and maxillofacial surgeries [1]. PL is biocompatible and has no risk of transmissible viral disease or prionic contamination. Platelet concentrates can be prepared by buffy coat or platelet-rich plasma (PRP) method [2], even from expired blood derivates. Freeze-and-thaw cycles, sonication, and thrombin/CaCl_2_ activation have been used to obtain PL [2]. The variability in PL manufacturing influences the content of main growth factors (GFs) that will be available in cell culture, such as PDGF isoforms, TGF-*β*1, and also VEGF, HGF, and bFGF. The inconsistency of the results makes PL functionality* in vitro* still not exhaustively explored. Furthermore, specific and optimal culture conditions will be searched [3, 4] especially for MSCs [5].

DPSCs received great attention for their osteogenic potential; besides they demonstrated an angiogenic potential [6], a commitment to melanogenesis [7], differentiation in neurons [8], and islet-like aggregates formation [9]. The transcriptomes and cytogenetical stability of DPSCs expanded under PL and FBS were previously compared and revealed similar profiles [10]. Also CFU-potential, the immunophenotype, and trilineage differentiation according to MSC requisites were comparable [10]. 5% PL was defined as the concentration for increased cellular proliferation and for good mineralisation* in vitro* for DPSCs [10–12]. In addition, the same concentration was used to seed DPSCs on biomaterials, showing positive effects on regeneration* in vivo* [11]. Often 10% PL inhibited the proliferation of DPSCs. Interestingly, DPSC cultures in PL had a higher ALP activity compared to FBS [11, 12]. We initially combined different assays to select an optimal PL concentration for DPSCs growth, by evaluating different cell health indicators, such as membrane permeability, cell division, and metabolic activity. The comparison between FBS and the selected PL concentration for mesenchymal stem cell capacity was evaluated by surface marker expression and gene expression of relevant transcriptional factors.

We focused on PL effects on repairing properties of DPSCs by performing* in vitro *migration and survival assay. Finally, we introduced PL in osteogenic and chondrogenic inducer media in order to evaluate its effect on DPSCs differentiation.

We characterised a standardised commercially available human allogenic platelet lysate as an attractive candidate for* in vitro* culture of DPSCs [10]. The use of this safe, quality-controlled, and potentially advantageous supplement could establish a preparatory study for regenerative medicine applications.

## 2. Materials and Methods

### 2.1. DPSCs Source and Cell Culture

Human-impacted third molars were extracted from 20-year-old healthy patients from Calabrodental Clinic (Crotone). All donors signed a written informed consent according to the Ethical Committee policy. To isolate DPSCs, the obtained normal teeth were washed in saline solution containing antibiotic solutions and immediately transferred to the cell culture laboratory. Mesenchymal stem cells were isolated under sterile conditions according to a previously published method [13, 14]. Briefly, pulp tissues were isolated and washed several times with PBS and further cut with a scalpel into small pieces. Subsequently, the whole small pieces were dissociated to have single cell suspension by enzymatic digestion, using 3 mg/mL type I collagenase and 4 mg/mL dispase (Sigma-Aldrich, Saint Louis) in Hank's Balanced Salt Solution (Invitrogen, Carlsbad). Then, samples were incubated for 1 h at 37°C in agitation and the digest was diluted in alpha-minimal essential medium (*α*-MEM, Gibco, Grand Island) supplemented with 10% FBS (Gibco, Grand Island) and centrifuged at 300 ×g for 5 min. The pellet was finally resuspended in fresh medium, seeded in culture dishes, and incubated at 37°C 5% CO_2_. DPSCs were routinely expanded in growth medium consisting of *α*-MEM supplemented at 10% FBS. Trilineage differentiation of DPSCs was early assessed and cell stocks were cryopreserved. Two donors were used for the study. PL was purchased from Sclavo Diagnostics (Sovicille).

### 2.2. Viability Assays

For live/dead imaging, DPSCs were grown on sterile cover slides 24 × 24 mm and cultured for 5 days in FBS or PL media. After formalin fixation, the cells were stained with a solution of Calcein AM-EthD-III (Biotium, Hayward) according to the kit indications. Epifluorescent signal was detected and relative images were acquired through SP5 microscope (Leica, Wetzlar). Metabolic activity of DPSCs was assessed by PrestoBlue (Molecular Probes, Eugene) assay. Briefly, cryopreserved DPSCs were thawed and resuspended in *α*-MEM containing FBS at 10% or PL at 5%, 2%, and 1%. Cells were seeded at 2500 cell/well in one 96-well plate for each analysed time point; more than 6 wells were replicated for each condition. An aliquot of fresh medium was provided to the plates that were cultured for more than 4 days. The PrestoBlue reagent was incubated as supplier instruction to a final volume of 100 *μ*L; after 2 hours the collected supernatant was measured for absorbance with Multiskan GO (Thermo Fisher, Waltham) spectrophotometer (570–600 nm). An additional control was set using human serum (HS) 10%. For viable cell counts, the samples were trypsinized after 3 days or 3 weeks and measured by ADAM-MC (AlphaMetrix, Rödermark) system to evaluate cell membrane permeability by dye exclusion.

### 2.3. Flow Cytometry

To investigate cellular proliferation, DPSCs were labelled with 5 *μ*M of 5-chloromethylfluorescein diacetate (CMFDA) in *α*-MEM for 45 min at 37°C CO_2_. Cells were washed in medium and seeded at 10^5^/well in a 12 well-plate, in duplicate, for different time points (2, 3, and 4 days). Cytoplasmic amount reduction of the dye was measured using NAVIOS flow cytometer (Beckman Coulter, Brea). The data were analysed with FlowJo software. For immunophenotype analysis, cells cultured for 1 week with 10% FBS or 1% PL were trypsinized and aliquoted in FACS tube. Cells were washed twice with PBS 0,1% BSA. To limit unspecific binding, a blocking step is performed by resuspension of the pellets with PBS 1% BSA for 15 min. Cells were stained on ice for 1 h with saturating concentrations of primary conjugated antibodies diluted 1 : 50 in PBS 0,1% BSA. CD13-PE (mouse IgG1), CD29-APC (mouse BALB/c IgG1), CD44-FITC (mouse IgG2b), CD45-APC-H7 (mouse IgG1), CD73-FITC (mouse IgG1), CD90-PE (mouse BALB/c IgG1), and CD105-APC (Mouse BALB/c IgG1) monoclonal antibodies purchased from BD (Franklin Lakes) and CD146-PE (mouse IgG1), CD34-FITC (mouse IgG2a), and HLA-DR-PE (recombinant human IgG1) monoclonal antibodies purchased from Miltenyi Biotec (Bergisch Gladbach) were used to define the MSC panel as previously described [15]. At least 10000 events were counted for each sample.

### 2.4. Real-Time PCR

To analyse cell differentiation state, gene expression was analysed as previously described [15, 16]. Briefly, RNA samples were obtained after extraction with PureLink RNA mini kit (Thermo Fisher, Waltham) following the manufacturer's instruction. Total RNA was quantified through spectrophotometry and 500 ng of RNA was subjected to reverse-transcription reaction using the High Capacity RNA-to-cDNA Kit (Applied Biosystem, Foster City). One microliter of cDNA was amplified by real-time PCR with the Power SYBR green PCR Master Mix (Applied Biosystem, Foster City). Real-time PCR reactions were carried out in a PikoReal 96 (Thermo Fisher, Waltham) apparatus with the following conditions: initial denaturation step at 95°C for 10 min, followed by 40 cycles of 10 s at 95°C and 1 min at 60°C. The specificity of PCR products was checked by analysis of melting curves. The expression of each gene was determined from the Ct value, and relative expression levels were calculated using the ΔΔCt method after normalisation to the expression of the HRPT housekeeping gene. All primer pairs sequences are listed in Table S1 in Supplementary Material available online at http://dx.doi.org/10.1155/2016/7230987.

### 2.5. Wound Scratch Assay

For scratch assay, DPSCs were seeded and grown until confluence in 35 mm dishes. An* in vitro* wound (600 *μ*m average size) was created in the monolayer by scraping it over the total diameter with a sterile 10 *μ*L pipette tip. Dishes were washed twice in *α*-MEM to smooth the scratch edges and remove any suspended cells. Cultures were refed with fresh complete medium containing 10% FBS or 1% PL. Reference markings were made on the external surface of the dishes to identify scraped zones to be photographed 2 hours and 24 hours after scratch. To quantify the wound healing capacity, images were manually analysed. A digital rectangle zone free of cells and centred on the wound breadth was set on 2-hour controls and thus was superimposed on the corresponding 24-hour images. Cell dense regions were drawn trough polygonal selection tool and measured by ImageJ software. Multiple selections were summed and healing was determined as percentage of the open (wound) area at 24 hours.

### 2.6. Chemotaxis Assay

An under-agarose method was adapted from Vogel et al. [17] Briefly, sterile melt 1% agarose in *α*-MEM was poured in 60 mm dishes. Three equally distant wells (≈5 mm) were made by pressing a sterile tip on the agarose gel surface. The gel was equilibrated overnight in *α*-MEM. DPSCs were harvested at exponential phase, washed in *α*-MEM, and seeded in the central well at concentration of 3.5 × 10^4^ cells/70 *μ*L. A volume of 70 *μ*L medium containing 10% FBS or 1% PL was added to the right well in order to create a chemoattractant gradient in culture, while a same volume of *α*-MEM was added to the left well as negative control of chemotaxis. After 24 hours of incubation at 37°C 5% CO_2_, both left and right wells were refilled of their content. At 48 hours, the dishes were gently washed and fixed in formalin 5% overnight at 4°C. Four pictures (10x zoom) were taken at 24 hours for quantitative analysis at each interwell's zone. The images were tiled and cell number was manually counted in ImageJ software. Only cells completely outside the well and under the agarose were considered. The counted number of migrated cells is corrected subtracting the number of cells which moved towards the negative control well.

### 2.7. Cell Survival Assay

The damaging effect of millimolar concentrations of H_2_O_2_ on DPSCs was tested in dose-dependent manner after 1-hour exposition in basal medium (BM) which consisted of *α*-MEM. PrestoBlue assay was performed after 48 hours to detect the challenge on viability [18, 19]; therefore an EC_50_ of 500 *μ*M was established for following experiments (Fig. S3). For cell survival test, DPSCs were seeded as 3.5 × 10^4^ DPSCs/well of a 96-well plate and left to adhere in complete media, 10% FBS or 1% PL. On the next day, all the wells were washed with PBS and 500 *μ*M H_2_O_2_ was added in basal medium (BM) or complete medium (CM) containing 10% FBS or 1% PL. Subsequently, cells were treated for 1 h in incubator. At the end of the treatment, H_2_O_2_ dilution was replaced with new complete medium. After 6 hours, at 37°C 5% CO_2_, PrestoBlue was incubated for 2 hours and its absorbance read. Alternatively, DPSCs were seeded in 12-well plates, treated as above, and trypsinized 6 hours later to perform viable counting by ADAM-MC.

### 2.8. *In Vitro* Osteogenic Differentiation

For osteogenic differentiation, growing cells were detached and seeded subconfluently in 60 mm Petri dishes. At 85–90% of confluence, cell medium was changed to osteogenic medium composed of *α*-MEM with glutamine (Gibco, Grand Island), 1% PL, 0,2 mM L-ascorbic acid-2-phosphate (Sigma-Aldrich, Saint Louis), 100 nM dexamethasone (Sigma-Aldrich, Saint Louis), 10 mM *β*-glycerophosphate (Sigma-Aldrich, Saint Louis), penicillin-streptomycin solution (Sigma-Aldrich, Saint Louis), and 0.25 mg/mL amphotericin B (Sigma-Aldrich, Saint Louis). The positive control group medium included 20% FBS in place of PL. Osteogenic induction was performed for 4 weeks, replacing media twice a week.

The differentiation was assessed by Alizarin red, quantified via spectrophotometry (405 nm) after dissolution in 10% acetic acid, and analysed by real-time PCR (primer listed in Table S1) for gene expression levels of osteogenic markers.

### 2.9. *In Vitro* Chondrogenic Differentiation

For chondrogenesis differentiation, DPSCs were initially detached and seeded subconfluently in 60 mm dishes. At 85–90% of confluence, growth medium was changed to chondrogenic medium composed of DMEM High Glucose (Gibco, Grand Island), 1% PL, ITS + 1 Supplement (Sigma-Aldrich, Saint Louis), 100 nM dexamethasone (Sigma-Aldrich, Saint Louis), 50 mg/mL L-ascorbic acid-2-phosphate (Sigma-Aldrich, Saint Louis), and freshly added 10 ng TGF-*β*1 (Miltenyi Biotec, Bergisch Gladbach). The positive control groups' medium did not include PL in the formula. Chondrogenic induction was performed for 3 weeks, replacing the media twice a week. Alcian blue staining was extracted in acetic acid and its relative quantification was performed by spectrophotometry (620 nm). For micromass culture 5 × 10^5^ cells were pelleted at 300 g and washed in 1 mL of medium free of TGF-*β*1 and then left to aggregate in incubator in 1 mL of chondrogenic medium (with TGF-*β*1). Chondrogenic induction was performed for 3 weeks, replacing the media twice a week.

### 2.10. Histology

In several wound scratch assays, the scraped dishes were fixed and stained with eosin for qualitative late time points (3 days and 7 days) analysis and storage of samples. For osteogenesis confirmation, the dishes were fixed in 10% formalin for 15 min, washed in distilled water, and stained with Alizarin red (5 mg/mL) for 30 min. The samples were then washed several times with distilled water until clarity. For chondrogenesis confirmation, wells were fixed in 10% formalin for 15 min, washed in distilled water, and stained with Alcian blue pH 2.5 (Bio-Optica, Milan) following the supplier's indications. Finally, the samples were washed twice in abundant distilled water. Chondrogenic micromasses were formalin-fixed overnight, embedded into agarose block, and processed for classical histology. Sections of 3,5 *μ*m thickness were prepared using microtome (Leica). The slides were finally stained with Alcian blue and counterstained in Azocarmine Red (Bio-Optica, Milan). All histological samples were photographed for qualitative analysis using optical microscopes and color cameras (Leica suite).

### 2.11. Statistics

All the plots were edited in GraphPad Prism software. The specific statistical method to interpret the graphs is described within the individual captions. Differences were considered statistically significant when *P* < 0.05. ^*∗*^ *P* < 0.05; ^*∗∗*^ *P* < 0.01; ^*∗∗∗*^ *P* < 0.001; ^*∗∗∗∗*^ *P* < 0.0001.

## 3. Results

### 3.1. Viability and Proliferation

**Figure 1:**
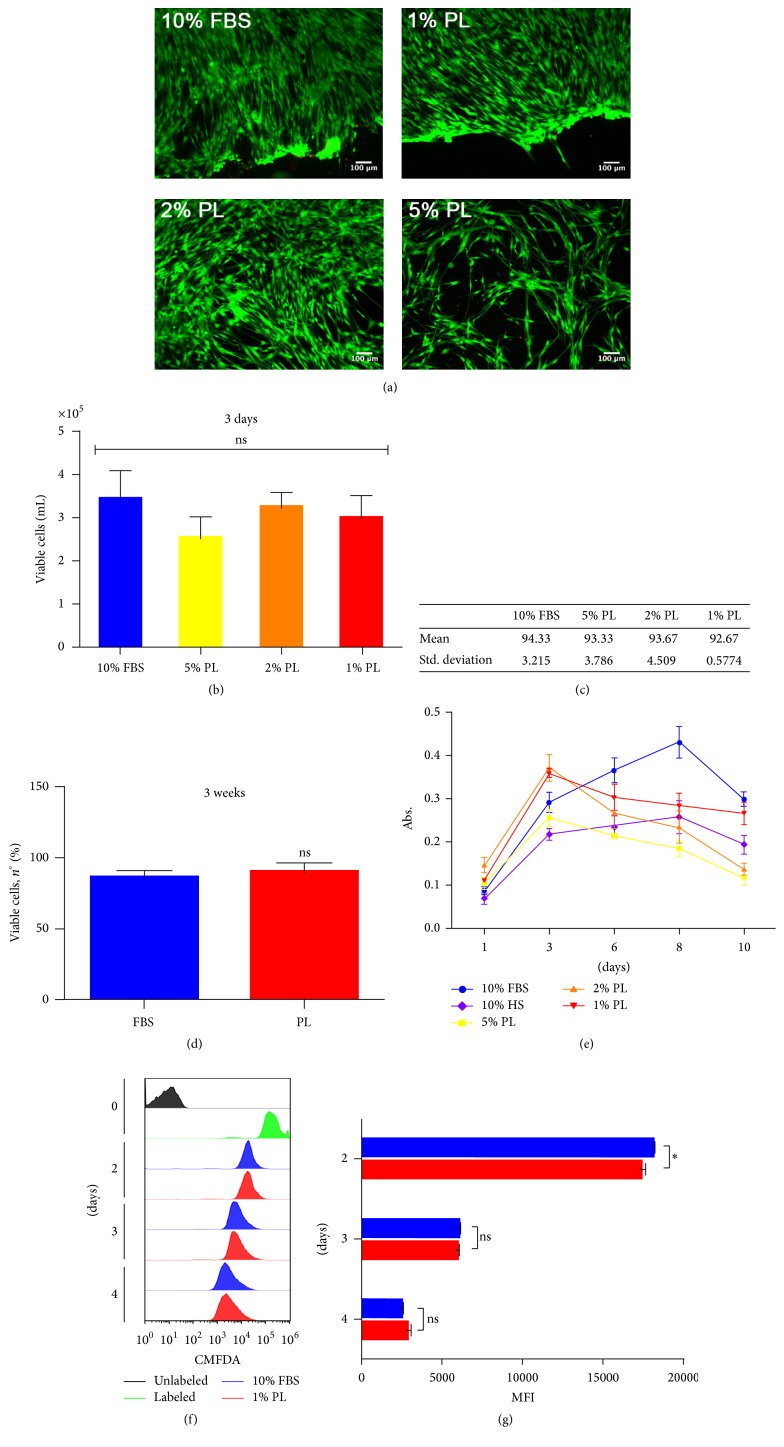
Effect of PL on the growth of DPSCs. (a) Representative images from live/dead fluorescence assay after 5 days of culture in different culture conditions. Green = live; red = dead. (b) Viable counts quantified by ADAM system after 3 days of culture. Data shown as mean + SD, *n* = 3, statistical significance according to 2-way ANOVA method (*P* < 0.05); ns: not significant. (c) Statistic values (average viability percentages) derived from samples in (b). (d) Viability after long-term culture of 3 weeks. Data shown as mean + SD, *n* = 2. ns: not significant according to unpaired *t*-test. (e) Cell growth activity measured by PrestoBlue assay. Data shown as mean + SD, *n* = 2. (f) CMFDA proliferation assay. Time-course comparing FBS and PL-selected condition. (g) Quantification of MFI derived from samples in (f). Statistics based on 2-way ANOVA method (^*∗*^ *P* < 0.05). ns: not significant.

The effect of different concentrations of PL ranging from 5% (high) to 1% (low) was initially compared to 10% FBS by observing viability parameters. The imaging of DPSCs, grown at high density for 5 days and marked with fluorescent live/dead staining, reported a high viability rate in all compared conditions, with only rare detection of dead cells across the whole sample (Figure 1(a)). Slightly morphological changes appeared in 2% (mid) and 5% (high) concentrations of PL, where long intercellular processes and circular association were visible at minor density. After 3 days of culture, no statistically significant difference among the conditions was shown, despite the fact that the average number of cells grown with 5% PL seemed to be lower (Figure 1(b)). Through relative viability quantification of the counted samples, we confirmed the live/dead staining qualitative results (Figure 1(c)). Viable counts of long-term cultures showed that 1% PL is still competitive in viability with respect to 10% FBS (Figure 1(d)). Different trends of metabolic activity were emphasised instead by PrestoBlue assay (Figure 1(e)). In particular, compared to FBS, both 1% and 2% PL showed a higher metabolic rate during the first days of culture, while the 5% PL never outdid FBS. Despite the fact that 1% PL and 2% PL showed a similar trend, 1% concentration brought fewer changes during the last days and presented more similarity to FBS controls until the plateau growth phase (10 days). To further assess if this low PL concentration was sufficient to promote DPSCs proliferation, cells within first days of culture were analysed by flow cytometry for the CMFDA tracer (Figure 1(f)). CMFDA labelling of DPSCs highlighted a significant higher rate of proliferation in 1% PL compared to 10% FBS during 48 hours from seeding (Figure 1(g)).

### 3.2. Stem Cell Properties

**Figure 2:**
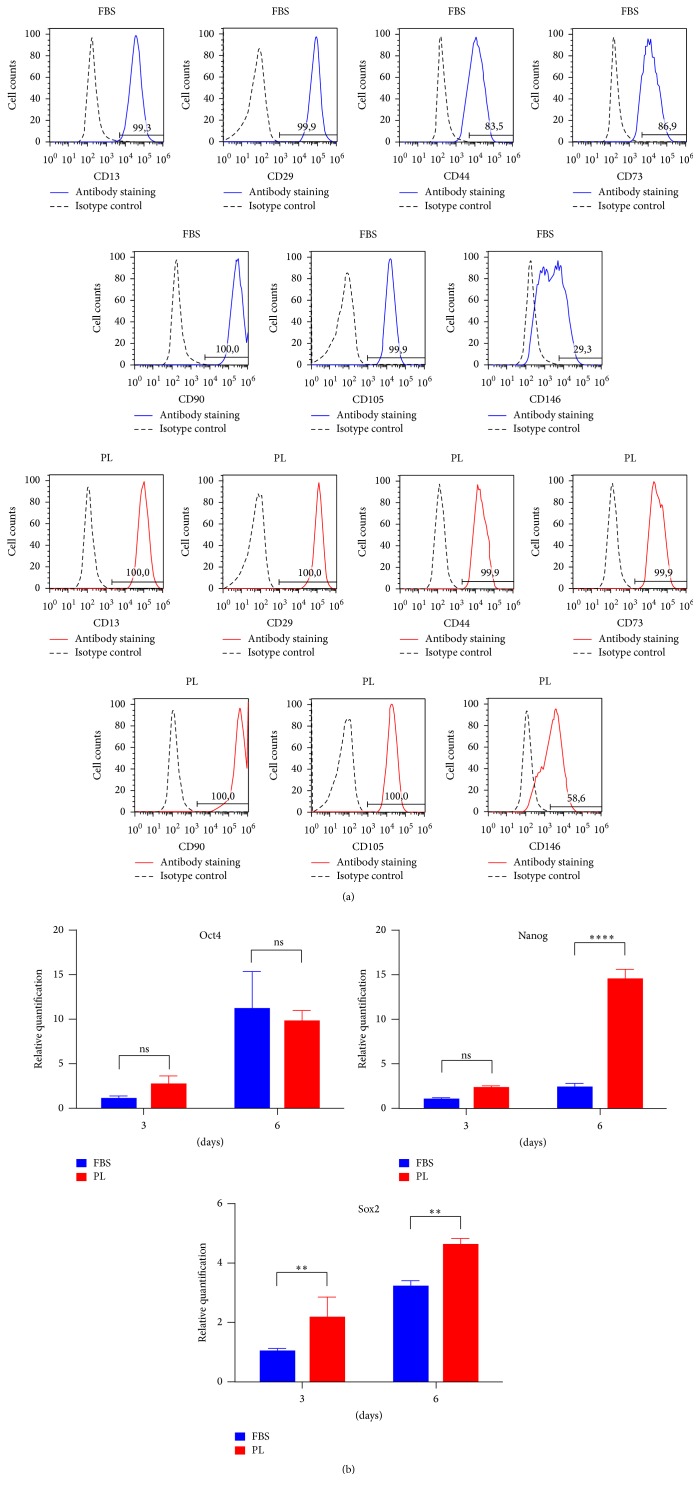
Stem cell markers comparison between FBS and PL cultures of DPSCs. (a) Immunophenotype for expressed MSC surface markers by flow cytometric analysis. The number specifies the percentage of gated cells positive for the indicated protein. Data representative of two experiments. (b) Relative levels of transcripts (fold change) for stemness markers derived from real-time PCR analysis. Statistical significance determined by 2-way ANOVA (^*∗*^ *P* < 0.05; ^*∗∗*^ *P* < 0.01; ^*∗∗∗*^ *P* < 0.001; ^*∗∗∗∗*^ *P* < 0.0001), mean + SD, *n* = 3.

To verify the identity of MSCs after the switch to 1% PL as supplement in cell medium, the DPSCs were analysed for MSCs surface markers by flow cytometry. The immunophenotype of DPSCs cultured in 1% PL resulted in coexpression of the obligatory ISCT markers [20] CD73, CD90, and CD105 and also the positive signal by CD13, CD29, CD44, and CD146 (Figure 2(a)), while it did not express negative markers such as CD34, CD45, and HLA-DR (Fig. S1). Intriguingly, the half population of DPSCs increased their CD146 expression when cultured in 1% PL. Compared to their controls, fluorescence geometric mean of PL is higher for CD13 and CD73 (data not shown). OCT4, Nanog, and Sox2 pluripotency markers were evaluated by quantitative PCR (Figure 2(b)). Samples cultured with PL until 6 days maintained the level of transcription of OCT4 compared to those cultured with FBS. On the other hand, PL supplementation timely increased Sox2 transcripts levels, while Nanog levels were significantly higher only at the last time point.

### 3.3. Migration Capacity

**Figure 3:**
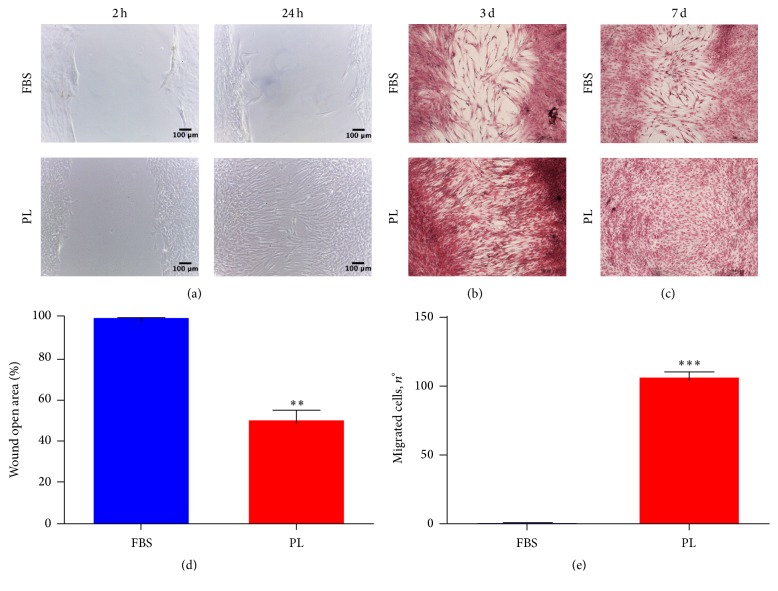
*In vitro* migration capacity of DPSCs is enhanced by PL. (a) Representative images of early time points during the scratch assay. Images were taken 2 hrs and 24 hrs after the initial scratch and are representative of 3 independent experiments. The cells were fixed and stained at different endpoints as shown in (b) for 3 days and (c) 7 days. (d) Quantitative analysis of wound healing at 24 hrs after scratch. 100% is set as open area 2 h time points. Data shown as mean + SD, *n* = 3. Statistical significance determined using unpaired *t*-test (^*∗∗*^ *P* < 0,1). (e) Quantification of chemotaxis derived from under-agarose assay. Data were collected using 24 h time points light microscope images. Data shown as mean + SD, *n* = 3. Statistical significance determined using unpaired *t*-test (^*∗∗∗*^ *P* < 0.001).

Platelet-derived GFs are involved in wound healing process [4]; thus we performed a scratch assay. Capturing images of different time points, we highlighted a greater level of wound closure for DPSCs scraped and then maintained in low-PL medium rather than in FBS medium. The enhanced cell migration in PL cultures from the wound edges is clearly evident 24 hours later (Figure 3(a)). Moreover, DPSCs cultured in low-PL medium continued to reduce the gap space after 3 and 7 days with a faster rate in comparison to FBS cultures (Figure 3(b)). One week after scratch, DPSCs in 1% PL were able to fully close the open wound area (Figure 3(c)). We also microscopically detected cell division in the wound space (Fig. S2), thus not restricting the repairing stimulation of PL to the migration of cells that were already present before the injury. Moreover, we counted the number of cells migrated at 24 h to have a quantitative result (Figure 3(d)). To address the question if this migrating ability can be associated with the soluble GFs contained in PL, we performed an under-agarose chemotaxis assay. After 24 hours, a conspicuous number of DPSCs positively responded to chemoattraction by PL, while very few cells migrated under the agarose gel to FBS containing compartment (Figure 3(e)).

### 3.4. Resistance to Cellular Damage Induced by H_2_O_2_

**Figure 4:**
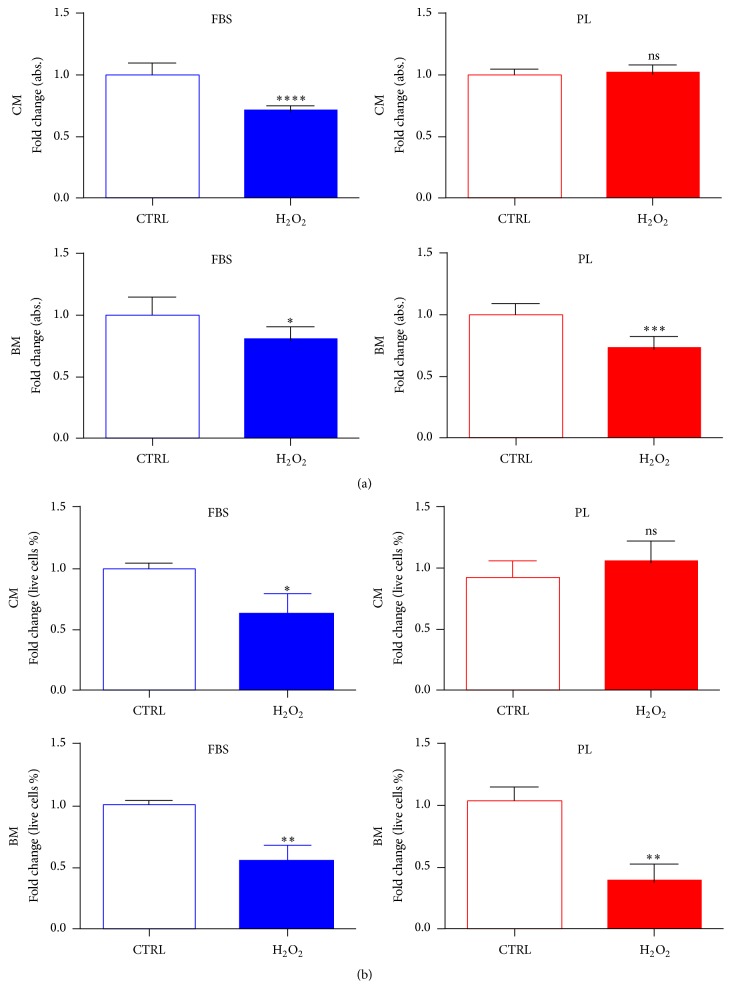
Impact of H_2_O_2_ on cellular viability is influenced by PL in culture. (a) Metabolic activity of cultures measured by PrestoBlue conversion 6 hrs after the treatment with H_2_O_2_. Data are expressed as mean + SD, *n* = 3, derived from 2 experiments. (b) Automatic viable cell counts after 6 hrs of treatment. Data were normalised to median value of control samples and shown as mean + SD, *n* = 3. To determine significant results, the unpaired *t*-test was performed (^*∗*^ *P* < 0.05; ^*∗∗*^ *P* < 0.01; ^*∗∗∗*^ *P* < 0.001; ^*∗∗∗∗*^ *P* < 0.0001). CM, treatment performed in complete medium; BM, treatment performed in basal medium.

The early effect on survival/recovery of cells treated with 500 *μ*M of H_2_O_2_ was analysed either by PrestoBlue assay (Figure 4(a)) or viable counting (Figure 4(b)) after 6 hours from the treatment. The presence in medium of PL before, during, and after treatment (complete media, CM) marked a nonsignificant difference output between untreated and treated samples, thus neutralising or rescuing the cells from the reactive oxygen species (ROS) action. On the contrary, the continuative presence of FBS in the wells (CM) was not sufficient to avoid cytotoxic effects of H_2_O_2_. Indeed, DPSCs conditioned in FBS or PL media but then exposed to H_2_O_2_ in basal media (BM) incubation reduced their vital activity measured by PrestoBlue reagent and were affected in own membrane integrity noticeable by dye exclusion cell counts.

### 3.5. Bony and Cartilaginous Differentiation

**Figure 5:**
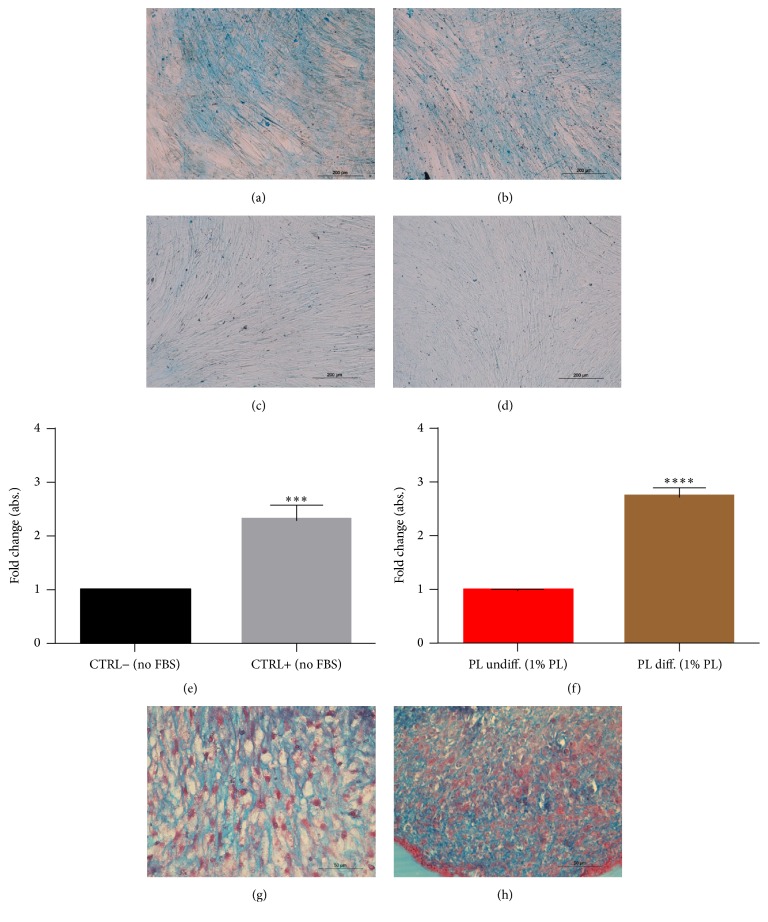
Effect of PL on chondrogenic differentiation of DPSCs. (a–d) Representative images of DPSC cultured for 21 days in chondrogenic medium without (a) or with (b) PL. They were positively stained by Alcian blue, while cells maintained in 10% FBS (c) or 1% PL (d) growth media were not. (e, f) Quantification of Alcian blue by spectrophotometry for assay controls (e) and samples containing PL (f). Significant levels according to unpaired *t*-test (^*∗∗∗*^ *P* < 0.001 and ^*∗∗∗∗*^ *P* < 0.0001). Data shown as mean + SD, obtained from three experiments. (g, h) Micromass culture of DPSCs showing positive histological staining for chondrogenesis for control (g) as well as the micromasses cultured with differentiating medium with 1% PL (h).

**Figure 6:**
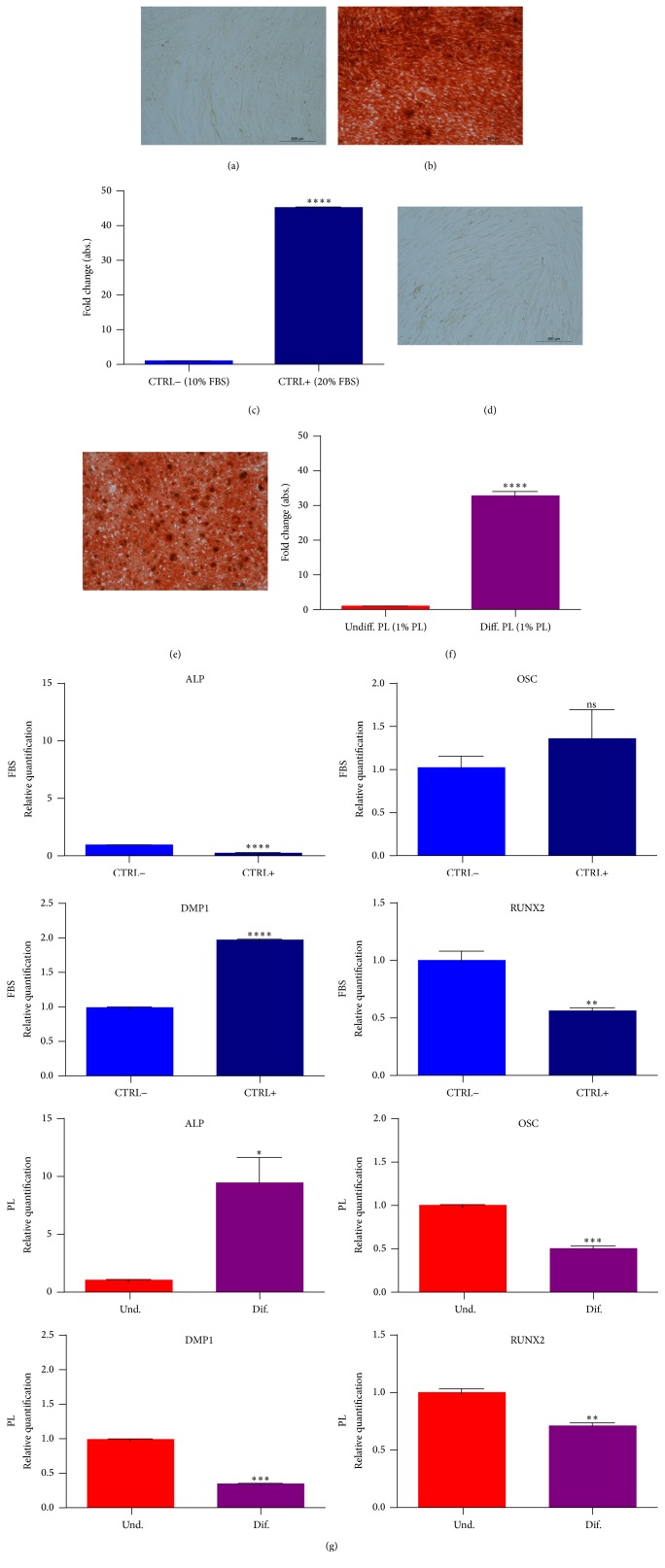
Effect of PL on osteogenic differentiation of DPSCs. Alizarin red staining on undifferentiated sample cultured in growth medium with 10% FBS (a) or 1% PL (d) and on samples differentiated with osteogenic medium containing 20% FBS (b) or 1% PL (e). Relative quantification of the same staining by spectrophotometry. Data shown as mean + SD, *n* = 3 samples. Two different experiments were performed. Significant levels according to unpaired *t*-test (^*∗*^ *P* < 0.05; ^*∗∗*^ *P* < 0.01; ^*∗∗∗*^ *P* < 0.001; ^*∗∗∗∗*^ *P* < 0.0001). (g) Relative quantification of osteogenic markers by real-time PCR on samples derived from the end (28 days) of the differentiating protocol. Data shown as mean + SD, *n* = 3. Significant levels according to unpaired *t*-test (^*∗*^ *P* < 0.05; ^*∗∗*^ *P* < 0.01; ^*∗∗∗*^ *P* < 0.001; ^*∗∗∗∗*^ *P* < 0.0001).

To test the suitability of PL for* in vitro* differentiation protocols, we basically substituted 20% FBS to 1% PL for osteogenesis of DPSCs; otherwise we added 1% PL to the serum-free formula for chondrogenesis. Results from Alcian blue staining showed the deposition of proteoglycans in both 2D (monolayers, Figures 5(a)–5(d)) and 3D conditions (micromasses, Figures 5(g) and 5(h)), confirming that PL did not impair chondrogenic differentiation of DPSCs. These results were confirmed by spectrophotometric measurement of Alcian blue complex formation (Figures 5(e) and 5(f)). Besides, FBS replacement with PL did not modify the formation of calcium deposits stainable by Alizarin red (Figures 6(a)–6(e)). Again, the differentiating condition in PL was statistically different compared to its respective undifferentiated control, as well as FBS condition (Figures 6(c) and 6(f)). Regarding genes analysis, PL samples showed at the end of differentiation a higher ALP expression compared to the respective basal state, a situation that did not correspond in FBS controls (Figure 6(g)). While FBS seemed to upregulate OSC and DMP-1 osteoblastic markers in 4 weeks in differentiating samples, we cannot affirm the same for PL differentiated cultures, in fact, showing lower relative levels at the same time of analysis (Figure 6(g)). RUNX2 transcription factor had a comparable fold increase in FBS samples as well as in PL samples (Figure 6(g)).

## 4. Discussion

So far, it has been observed that the proliferation rate of MSCs cultured in PL-supplemented medium was higher than that of those cultured in FBS-supplemented medium [21, 22]. The used concentrations of PL usually ranged from 10% to lesser than 1%, but the great heterogeneity of the results, mostly due to the preparation of PL, still delays the finding of the best concentrations to be used for specific cell type and application. The highest percentages, for example, 10% PL, often were not suitable to culture or expand the cells [11, 12], while 5% PL was the most tested and promising concentration for MSCs [23–26]. Here, we observed a good viability for 5% PL cultures but at the same time we noted resistance to trypsinization at this concentration. Partially, in accordance with other reports [27, 28], the DPSCs had easier and faster detaching from the plate compared to FBS when the concentrations were lower than 5% PL. At 1% PL, we could see that DPSCs attached quicker than FBS after cell seeding. Using the same low-PL concentration, DPSCs displayed a good viability and proliferation profile. Already Lee et al. showed the feasibility of low-percentage supplementation of PL for DPSCs growing and osteogenic differentiation [29]. On the base of our results, we choose 1% PL as selected concentration to adopt in culture for characterising DPSCs stemness, multipotency, and role in repair.

The immunophenotype of DPSCs cultured in PL was completely faithful to MSCs markers panel, confirming previous studies by colleagues [10]. Many stem cell lines express CD13; among the MSCs sources it is particularly present in oral tissues, while it is absent in bone marrow compartment [30]. The lack of this receptor was shown to impair* in vitro* adhesion to several ECM proteins, migration, and invasion [31]. Our PL cultures of DPSCs consistently expressed CD13. CD13 together with CD29 and CD73 [32] and CD44 [33] were associated with enhanced migrating phenotype of MSCs. CD146 is an endothelial and pericyte marker [34]. It is known to be downregulated in culture with FBS [35]. Moreover, our DPSCs expressed the CD146 receptor on their membrane when cultured in PL in comparison to FBS. This agrees with recent data about CD146 increased expression by BM-MSC in PL medium [24]. Both CD13 and CD146 are angiogenic markers; thus our study generated preliminary data to further include DPSCs angiogenesis protocols. Embryonic stem cell markers, in particular transcription factors such as Oct4, Nanog, and Sox2, were usually thought to have a similar role in maintenance of stemness also in DPSCs [36–38]. In general, they are indicative of a more immature phenotype for MSCs [39]. Nonetheless, it is very hard to understand the impact of their transcripts levels in DPSCs; an increase of their expression sustains a proliferating condition by cells. Our DPSCs cultures in PL sustained a higher gene expression of Sox2 [40], which also plays a role in migration of DPSCs [41].

MSCs can be exposed to various chemical or physical stresses, which may induce a cellular decay. MSCs [42] and HSCs [43] share with leucocytes the capacity of homing to damaged sites in several tissues. It is essential that DPSCs will move to the site of new skeletogenesis or during MSC-mediated tissue repair. Many migration assays are available tools for oral regeneration studies [44]. PL was also already described as efficient in promoting* in vitro* wound healing of dermal fibroblast [45], C2C12 mouse myoblasts [46], keratinocytes [47], and other human cells [48]. So far, an indirect enhanced migration and chemotaxis by BM-MSCs were induced by coating of platelet lysate on HA/b-TCP scaffolds [49]. In response to the injury, the DPSCs cultured in PL improved their ability to fix an induced mechanical damage such as a wound. In addition, we demonstrated that PL had a superior chemotactic activity than FBS, a reason which is likely correlated to the better migration of DPSCs towards the wounded region. Even though our experimental setting aimed to show how the healing of a scratched monolayer of DPSCs involved just the migration mechanism, surprisingly also cell genesis was active during the reparative process. Oxidative stress can lead to a wide range of cellular damage, such as damage to membrane lipids, proteins, and DNA. It is widely accepted that increasing concentration of free radicals augmented premature senescence even* in vitro* [50] or associated with bone disorders [51]. The platelets are very reactive* in vivo* against H_2_O_2_ which is a modulator and an efficient inducer of their aggregation [52]. SOD enzyme, also derived from the platelets, inhibited neutrophils excessive production in reactive oxygen species (ROS). Despite the role of platelets in releasing free radicals, their granules contained also the enzyme catalase. Our experiments supposed the rescue of DPSCs treated with H_2_O_2_, implying a neutralization action (supported by cell membrane permeability data) or high-level metabolic recovery (mostly mitochondrial). We speculate that the quite antioxidant activity detected with PL could mainly derive from catalase residues in the preparation and may be a beneficial protecting feature for MSCs, if this supplement is used. However, it is not possible to exclude that the less susceptibility to H_2_O_2_ is an intrinsic capacity that the cells acquire for the adding of PL. Nonetheless, a deeper comprehension would be desirable; this effect of PL we showed was not observable at the same level for FBS.

Moreover, PL is highly requested for cell-based therapies such as tissue engineering and bone and cartilage regenerative approaches [53]. Chondrogenic and osteogenic potentials were broadly studied and they were retained and influenced by PL added in cultures of MSCs [21, 54, 55]. MSCs cultured in 5% PL were seen to spontaneously activate osteoblastic gene expression [56]. Many authors documented that appropriate concentrations (i.e., 5% PL) enhanced, in particular, DPSCs mineralised differentiation and showed odontogenic potency [11, 12, 24]. The generation of protocols aimed at chondrogenesis taking advantage of PL is an expanding field [10, 21, 25, 57, 58], because PL is a rich source of natural TGF-*β* and the regenerative potential of primary chondroblasts is more restricted. PL was recently implemented in scaffolds construction [59] and scaffolds functionalization procedures [49]. We assessed the compatibility for the low-percentage concentration of commercial PL to be introduced in osteogenic and chondrogenic traditional protocols for DPSCs. Indeed successful colorimetric detection was measured either for calcium deposits by Alizarin or for proteoglycans by Alcian blue. We found an active gene transcription of the two osteoprogenitor markers ALP and RUNX2. Even if a total improvement in osteogenesis after the supplement comparison cannot be assumed at the selected concentration, the objective advantage of a xeno-free condition outlines an optimal reason to switch for our research-grade protocols in which GFs are delivered from PL. Actually, the positive controls conditions for differentiation were considerably different from testing conditions. Indeed, a 20% FBS and 1% PL, for the osteogenesis, were compared. Respectively, the presence of PL during chondrocytic development was used instead of absence of serum, suggested from the historical protocol [60].

Further studies will helpfully evaluate the feasibility of the use of PL in DPSCs isolation as already described for BM-MSC [23, 54, 61]. Other elucidations are required for the immunomodulation function in the presence of standardised PL preparations, like the one tested in this work. So far, the dual combination of PL with platelet-poor plasma (PPP) was shown to be particularly efficient to boost the number of clonogenic precursors from tissue biopsies, that is, primary cultures of MSCs from bone marrow, umbilical cord, and adipose tissue [62, 63].

## 5. Conclusion

Taken together, our results suggest that human allogenic PL is a suitable alternative to FBS for expansion and differentiation of DPSCs* in vitro*. Furthermore, up to our knowledge, this is the first study reporting the influence of PL on DPSCs migration and antioxidant effect during* ex vivo* maintenance.

## Supplementary Material

The supplementary material provides the immunophenotype comparison for negative MSC markers, the images of cell growth occurring during in vitro wound closure, the dose response cytotoxic effect of H_2_O_2_, the primer list used for RT-qPCR experiments.
