# Supplementary material for: Highly Efficient In Vitro Reparative Behaviour of Dental Pulp Stem Cells Cultured with Standardised Platelet Lysate Supplementation
Source: Stem Cells Int. 2016 Sep 28;2016:7230987. doi: 10.1155/2016/7230987 (PMC5059612; doi:10.1155/2016/7230987)
Supplement: Supplementary file 1 — The supplementary material provides the immunophenotype comparison for negative MSC markers, the images of cell growth occurring during in vitro wound closure, the dose response cytotoxic effect of H2O2, the primer list used for RT-qPCR experiments. [file 7230987.f1.docx]

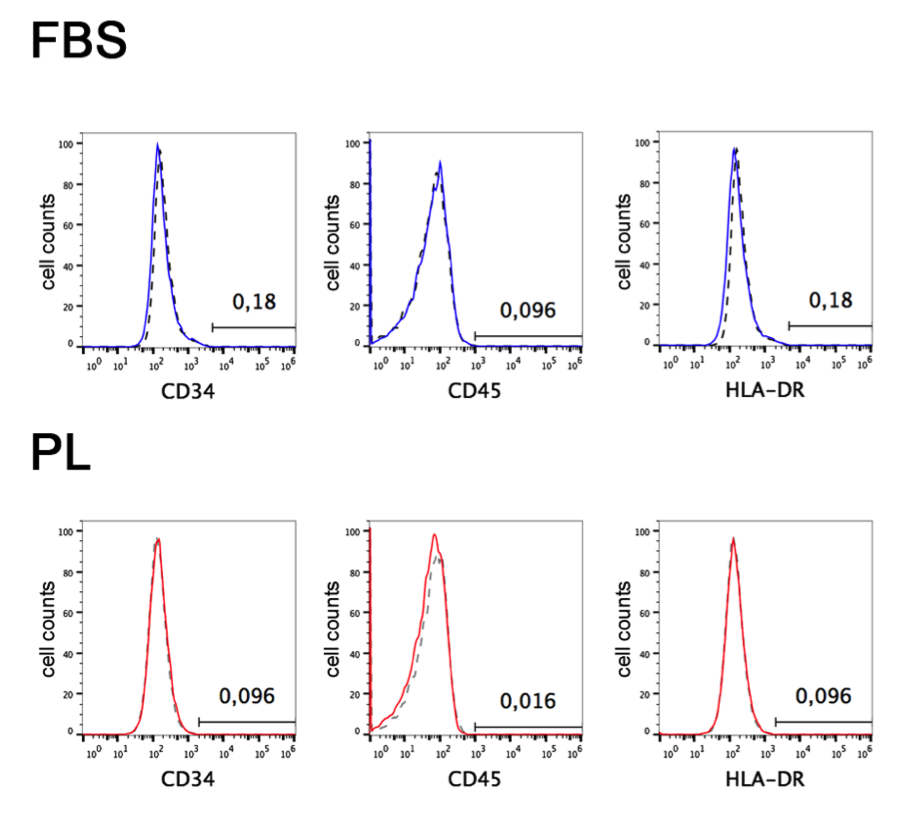


Fig. S1 Immunophenotype comparison for negative MSC markers. Both 10% FBS and 1% PL cultures of DPSCs showed the absence of the selected markers associated to a non-MSC phenotype. Dashed line; isotype control. Continued color-line: antibody staining


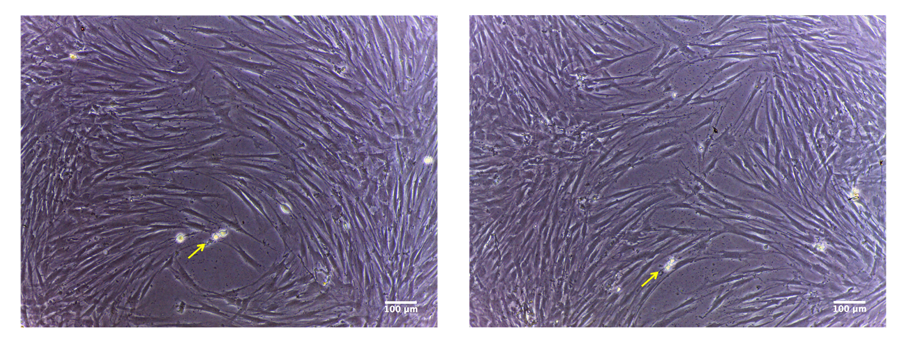


Fig. S2 Cell growth occurring during *in vitro* wound closure. Yellow arrows indicate mitosis in DPSCs cultures maintained in α-MEM 1% PL. Optical microscopy captures, 24 hrs post the initial scratch.


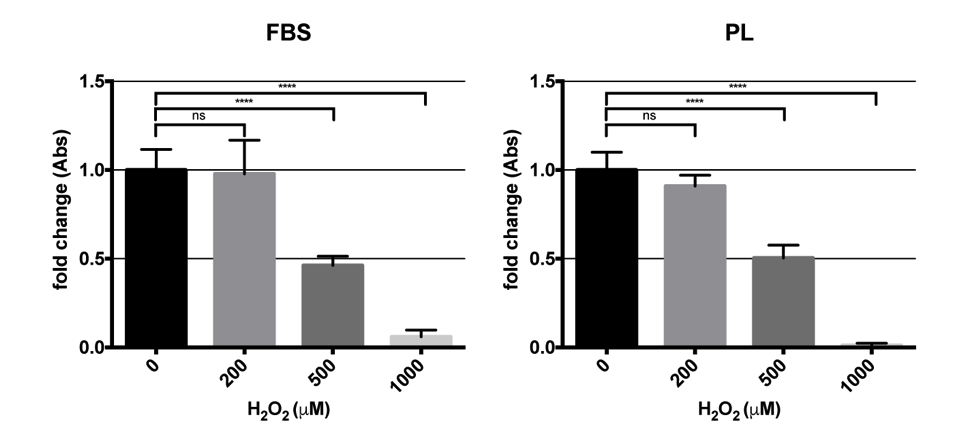


Fig. S3 Dose response cytotoxic effect of micromolar concentrations of H_2_O_2_. DPSCs were incubated in basal medium (BM) prived of FBS or PL for 1h 37°C CO_2_ then viability was measured by Prestoblue reagent after 48 hrs. 500 µM was chosen as effective concentration (EC_50_) for cell resistance evaluation.

Table S1. Primers used for RT-qPCR experiments.

| Gene | Primer | NCBI ref. |
| --- | --- | --- |
| ALP | FW: TAAGGACATCGCCTACCAGCTC | NM_000478.4 |
|  | RV: TCTTCCAGGTGTCAACGAGGT |  |
| DMP1 | FW: GTGAGTGAGTCCAGGGGAGATAA | NM_004407.3 |
|  | RV: TTTTGAGTGGGAGAGTGTGTGC |  |
| NANOG | FW: ATTCAGGACAGCCCTGATTCTTC  RV: TTTTTGCGACACTCTTCTCTGC | NM_024865 |
| OCT4 | FW: GTATTCAGCCAAACGACCATC  RV: CTGGTTCGCTTTCTCTTTCG | NM_002701 |
| OSC | FW: TGAGAGCCCTCACACTCCTC | NM_199173.4 |
|  | RV: ACCTTTGCTGGACTCTGCAC |  |
| RUNX2 | FW: ATGTGTGTTTGTTTCAGCAGCA | NM_001024630.3 |
|  | RV: TCCCTAAAGTCACTCGGTATGTGTA |  |
| SOX2 | FW: GACTTCACATGTCCCAGCACTA  RV: CTCTTTTGCACCCCTCCCATT | NM_003106 |
